# Occurrence of Isopods in Two Species of Snappers (Lutjanidae) from Northeast Brazil

**DOI:** 10.1155/2019/8176283

**Published:** 2019-05-02

**Authors:** André M. Alves, Marina G. Leonardo, Geza T. R. Souza, Ricardo M. Takemoto, Flávia S. de Lima, Luiz E. R. Tavares, Cláudia M. Melo, Rubens R. Madi, Verónica L. S. Jeraldo

**Affiliations:** ^1^Laboratório de Biologia Tropical, Instituto de Tecnologia e Pesquisa, Universidade Tiradentes, Aracaju, Sergipe, Brazil; ^2^Instituto Federal de Educação, Ciência e Tecnologia de São Paulo, Avaré, São Paulo, Brazil; ^3^Núcleo de Pesquisas em Limnologia, Ictiologia e Aquicultura, Laboratório de Ictioparasitologia, Universidade Estadual de Maringá, Maringá, Paraná, Brazil; ^4^Centro de Ciências Biológicas e da Saúde, Universidade Federal do Mato Grosso do Sul, Campo Grande, Mato Grosso do Sul, Brazil

## Abstract

Parasitic isopod species are poorly known in the northeastern coast of Brazil. In this sense, this study presents novel records of Isopoda of the families Aegidae, Cymothoidae, and Corallanidae. A total 69 specimens of* Lutjanus analis* and 19 of* Lutjanus jocu*, of which 46 isopods from 3 different species were collected, i.e.* Rocinela signata*,* Cymothoa excisa,* and* Excorallana richardsoni*. The species* R. siganata* and* E. richardsoni* are reported for the first time in* L. jocu*. A significant relationship between the parasite* R. signata* and the size of the fish* L. jocu *was also observed. The isopod* C. excisa* is considered an incidental finding in* L. analis*. To the authors' knowledge, this is the first report of this species in fish from Brazil. The three species of isopods are new occurrences in the State of Sergipe, northeast region of the country. An additional morphological characteristic observed in the dorsal setae of pleotelson in specimens of* E. richardsoni *was that one end of this structure wws bifid. This information contributes to the current body of knowledge of the morphology of this particular species.

## 1. Introduction

Lutjanidae is a family of cosmopolitan fish that occur most often in regions of tropical and subtropical seas of Australia, Africa, North America and South America. In Brazil, several species of this family are widely explored by artisanal fishermen. Because of their generalistic carnivorous behavior, they play an important role in the ecological control of other populations in reef environments of coral reefs. On the Brazilian tropical coast, 15 species of fish belonging to Lutjanidae have been reported including the “Cióba” -* Lutjanus analis* (Cuvier, 1828) - and the “Dentão” -* Lutjanus jocu* (Bloch & Schneider, 1801). These two species are important fish resources in the State of Sergipe, northeast region of the country [1–7]. Besides their carnivorous and generalist habits, these fish also have a migratory behavior which may contribute to the occurrence of ectoparasitic infestastions such as crustacean infestation. Crustaceans are the group of ectoparasites with the largest morphological variety and diversity. It is estimated that there are approximately 5.400 species of parasitic crustaceans which include the isopods [8]. To date, more than 9.000 species of isopods have been reported in the literature. They are divided into free-living forms and parasitic forms. Species that have parasitic habits may be fixed in body sites of fish such as the gills, integument, and oral cavity. In South America, several species of isopods have been recorded [9–14]. However, the fauna of parasitic isopod species is not yet fully known in the northeastern coast of Brazil which warrants studies. In this sense, the present study aimed to report new occurrences for isopods Aegidae, Cymothoidae and Corallanidae and to bring a morphological description of isopods collected from lutjanid from the coast of northeast Brazil.

## 2. Materials and Methods

For this survey, fish were purchased monthly according to the supply and availability at the Municipal Fishing Terminal of Aracaju, SE, Brazil (10°54′17′′S 37°2′56′′W) between March 2015 and October 2016 directly from artisanal fishermen working along the northeastern coast of Brazil in the Western Atlantic. These fish were shipped to our laboratory and identified at the species level according to the taxonomic keys published by [1]. Parasites from these fish were identified according to the keys provided by Menzies and Kruczynski [15], Brusca and Iverson [16], Thatcher et al. [17], Cardoso et al. [11], and Silva and Souza-Filho [18]. The terminology used was based on Kensley and Schotte [19]. Total length, weight, and parasitological analysis of fish were assessed according to the protocols published by Eiras et al. [20]. Prevalence (p) in percentage and mean intensity (im) were calculated according to guidelines provided by Bush et al. [21]. The correlation coefficient “rs” by Spearman posts was used to determine possible correlations between total host length and parasites abundance. Some specimens of isopods had their appendages removed, mounted on glass slides, and examined under the light microscope. The illustrations of the parasites prepared using a Coleman microscope (model N-120) with a light-coupled camera. Representative specimens were deposited at the Museum of Zoology of the State University of Campinas (ZUEC) - Unicamp, Campinas, SP, Brazil.

## 3. Results and Discussion

A total of 88 fish, 69 specimens of* L. analis,* and 19 specimens of* L. jocu* were analyzed, of which 13.8% were parasitized by at least one species of isopod. We found a total of 46 specimens of parasites which belonged to the families Aegidae, Corallanidae and Cymothoidae.* Cymothoa excisa* Perty, 1833 (males, females, and juveniles),* Rocinela signata* Schioedte & Meinert, 1879 and* Excorallana richardsoni *Lemos de Castro, 1960 were the species of parasites from these three families that were found in the fish examined.* R. signata *was the species of greater prevalence in both* L. analis* and* L. jocu*, followed by* E. richardsoni* and* C. excisa* (Table 1).

The species* Cymothoa excisa* was found in a juvenile* L. analis*. There was a positive and significant correlation between the length of* L. jocu* and the abundance of the parasite* R. signata* (p = 0.0026). The parasite was found in males and females of two host species:* L. analis* - 4 isopods in two male specimens and in* L. jocu* - 6 isopods in 5 male specimens and 1 isopod in 1 female specimen. A male of* L. jocu* had 2 species of isopods in its gills:* R. signata* and* E. richarsoni*.

The following are the morphological characters and taxonomy of the specimens found in the fish examined:

Aegidae Leach, 1815

*Rocinela* Leach, 1818

**Figure 1:**
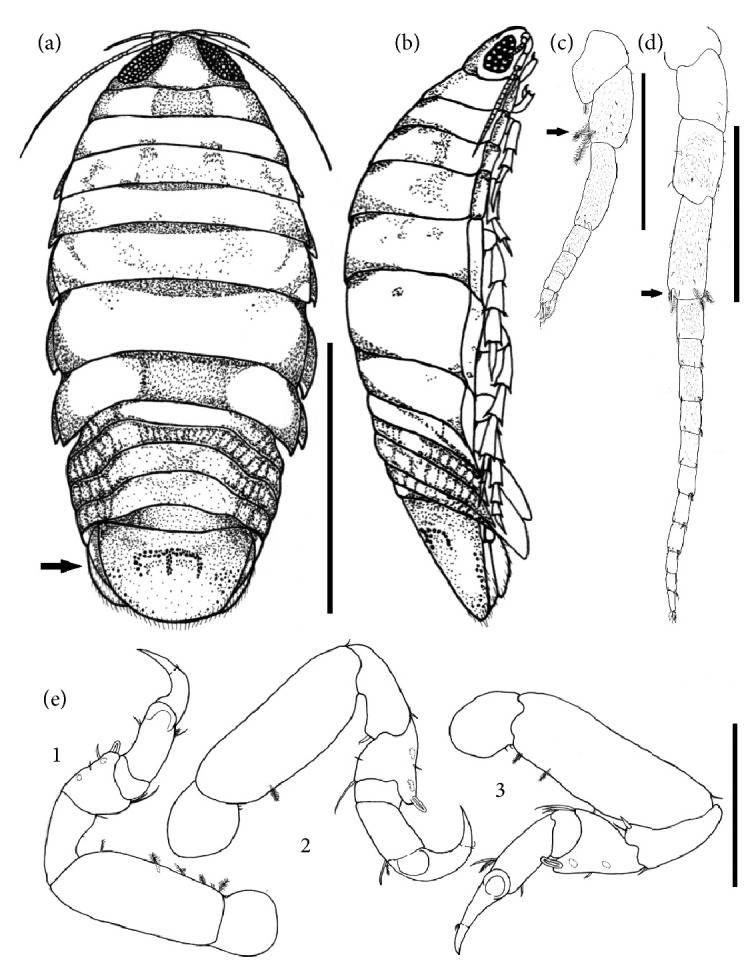
(a) Illustration of* Rocinela signata* collected from specimens of* Lutjanus analis* and* Lutjanus jocu*. Arrow shows the pleotelson with the typical mark of “inverted W”. Bar: 5 mm; (b) lateral view; (c) Detail of antenna I. Bar: 0.6 mm; (d) Detail of the antenna II. Bar: 0.7 mm. Arrows in (b) and (c) show the feathery bristles of the antennae. (e) Detail of the first three pairs of legs, bar: 0.4 mm.

*Rocinela signata* Schioedte & Meinert, 1879 (Figure 1)

Morphological description: Body slightly arched, cephalothorax evident and well-detached, fused to the 1st somite but not inserted to it, and previously triangular. It has two large, prominent, spaced eyes. Pereon with 7 somites, the 5th, 6th, and 7th being the largest and widest ones. Antenna tapering with 15 segments, the 4th being the longest one. In the distal region of this structure, there are 5 large and feathery setae (Figure 1(c)) and a flagellum with 11 segments. Antellunae smaller than the antenna (Figure 1(b)) with 7 segments, the 1st being the most robust and the 3rd the longest one, flagellum with four segments. The inner distal region of the 1st segment has a feathery bristle. In its inner face, near the distal region of the 2nd segment, there are five feathery setae - three larger setae, two smaller setae, and four segmented setae. Pereon with eight pereonites and coxal plates. The pleon has five somites. It has seven pairs of pereopods with single branches which end in scythe, pereopods 1-3 (Figure 1(e)) with a single spine on the posterior margin of the propodium. The telson has an inverted “M” or “W” shape on its dorsal surface which is typically found in this species (Figure 1(a), arrow).

Host:* L. analis, L. jocu.*

Infestation site: Gills (Gill filaments)

Location: Northeastern Coast, Aracaju, SE, Brazil.

Cymothoidae Leach, 1818

*Cymothoa* Fabricius, 1793

**Figure 2:**
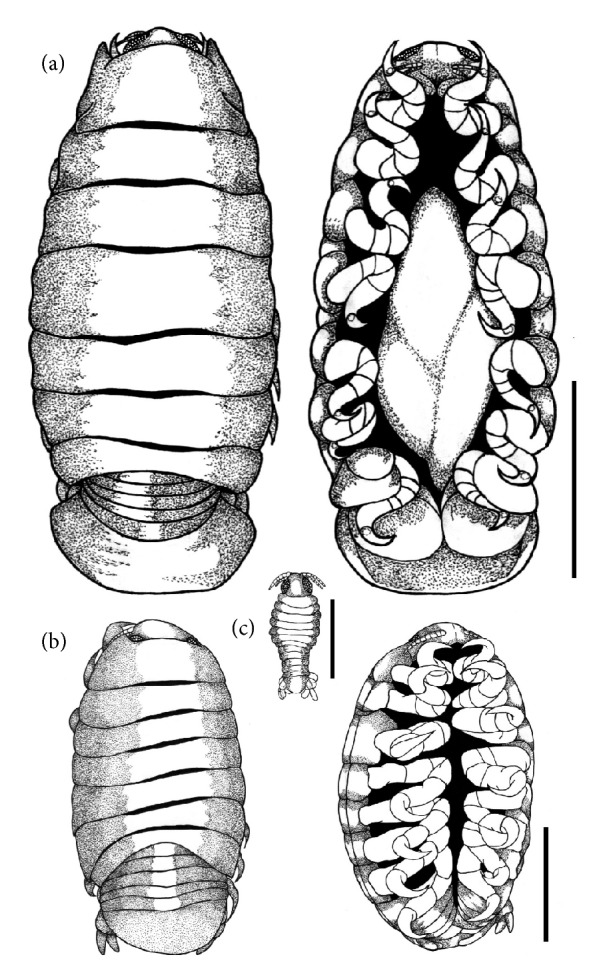
*Cymothoa excisa* collected from* Lutjanus analis*. Dorsal and lateral views of the female, respectively. Bar: 10 mm; (b) Dorsal and ventral views of the male. bar: 2 mm. (c) Dorsal view of manca. Bar: 0,02 mm.

**Figure 3:**
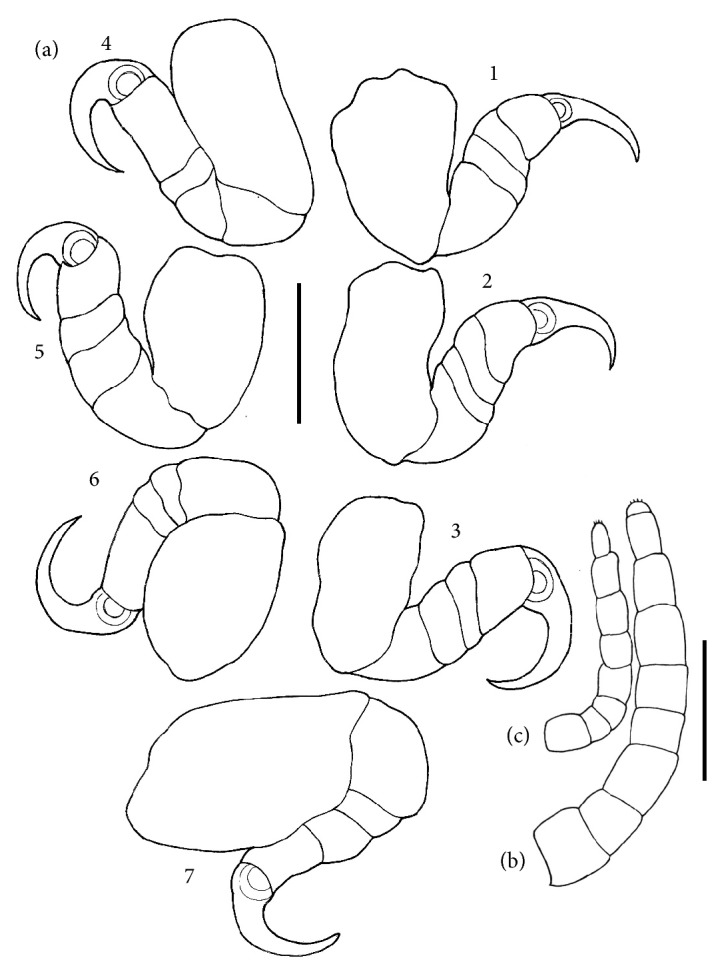
Pereopods of* Cymothoa excisa* collected from* Lutjanus analis*, 1-7 represent pairs of perionites that end in strong prehensile claws. Bar: 1 mm. (b) and (c) antenna and antennula, respetively, bar: 0.5 mm.

*Cymothoa excisa* Perty, 1830 (Figures 2 and 3)

Morphological description: Large and truncated body in the anterior region. Partially convex pereon. Cephalon distinct, not lobed, and deeply immersed in the 1st pereonite, with anterolateral margins surrounding the cefalon. Traces of eyes present and visible, but not prominent (Figure 2(a)). Antenna bases are expanded and well-separated. Antenna has nine segments. Antennula smaller than the antenna, with eight segments. Antero-lateral angles of the 1st sub-acute pereonite. Segments os Pleon (pleonites) to a certain extent are narrower than the pereonites and are deeply immersed in the 7th pereonite. Coxal plates are not strongly expanded, not reaching the edges of the pereonites. Seven pairs of pereopods divided into six segments each, all of which ending as prehensile claws (Figure 3). Male (Figure 2(b)): Body similar to the female's body, but smaller, having finer structures with few differences compared with those of the female. The juvenile (Figure 2(c)), in the post-mancas stage, morphologically identical to adults, but in smaller size and already presented the last thoracic segment or 7th pereonite and 7th pair of pereopods still absent.

Host:* L. analis.*

Infestation Site: oral cavity (tongue)

Location: Northeastern Coast, Aracaju, SE, Brazil.

Corallanidae Hansen, 1890

*Excorallana* Stebbing, 1904

**Figure 4:**
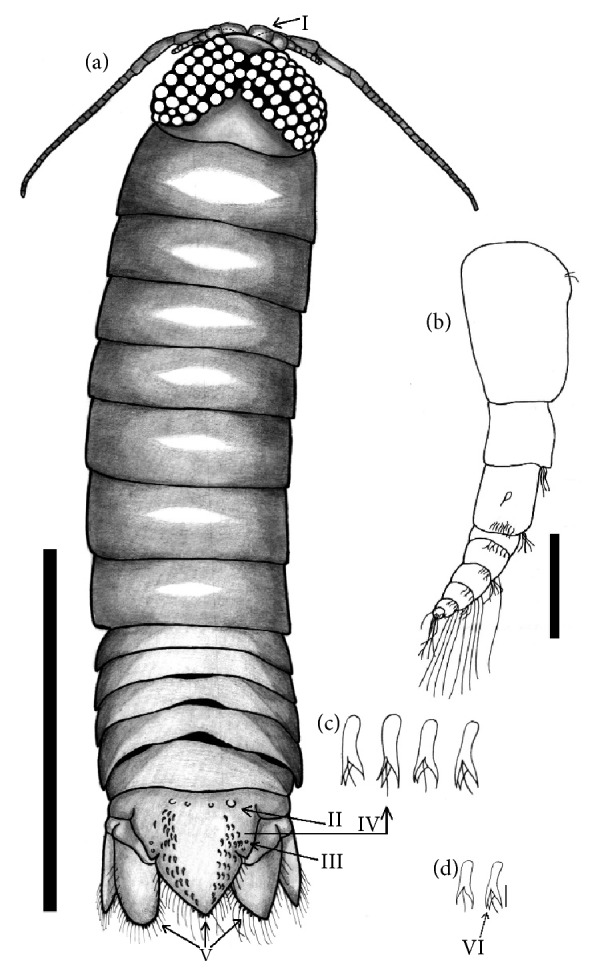
*Excorallana richardsoni* collected from* Lutjanus jocu*, dorsal view. Bar: 2 mm; (b) Antenna I. Bar: 0.2 mm; (c) Detail of the bristles. Arrow I - Swollen basal segment of the first antenna; setae II and III - Tubers of the anterior and lateral pleotelson, respectively; Arrows IV and V - position of the pleotelson bristles and detail of these structures; (d) Comparison between sow bugs described by Menzies and Kruczynski [15] and those described in the present study, arrow points to the bifid end of the sow bug.

**Figure 5:**
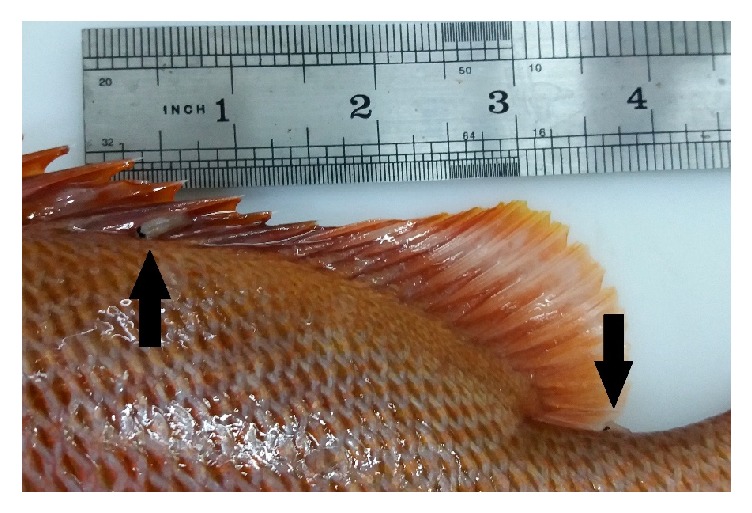
*Excorallana richardsoni* adhered to the body surface of* Lutjanus jocu* acquired from artisanal fishermen between the years of 2015 and 2016.

*Excorallana richardsoni* Lemos de Castro, 1960 (Figures 4 and 5)

Morphological description: Body quite elongated, longer than wide (Figure 4(a)), and slightly arched. Eyes: large, well prominent and contiguous, but do not extend to the anterior margin. Cephalon without tubers. Antenulla (Figure 4(a), highlight of arrow I, Figure 4(b)) smaller than the antenna. Flagellum formed by 6 peduncle segments. Antenna extends and reaches up to pereonite 2 and has 24 peduncle segments forming the flagellum. Six pairs of segmented paws with bristles and spines. The paws end in a scythe as the distal segment. The pleotelson is triangular with a sharply rounded apex, and the anterior region has 4 tubers (Figure 4(a), arrow II). The pleotelson has 2 incisions, one on each side, and there are two small tubers pin front of each incision (Figure 4(a), arrow III). Pleotelson has two setose rows in the dorsal region (Figure 4(a), arrow IV) spaced with bifid setae, with midapical filamentous setae. One end of each setae is bifid (Figure 4(c)). The same bifid setae are present at the apex of the pleotelson and uropods, Uropods with fringes of long feathery setae and slightly longer than the apex of pleotelson,

Host:* L. jocu*.

Site of Infestation: Buccal cavity, Gills, Body surface.

Location: Northeastern Coast, Aracaju, SE, Brazil.

Lima et al. [22] recorded isopods in* Scomberomorus brasiliensis* Collette, Russo & Zavala-Camin, 1978 and found* R. signata* in 31% of parasitized fish. Other authors also reported the presence of the parasite in Lutjanidae (snappers) including Cavalcanti et al. [23] that recorded* R. signata* in* L. synagris* with 10% of parasitized fish and a mean intensity of 1.25. The parasite has a distribution from Central America to South America, and has been recorded by Hermida et al. [24] in* L. analis*, corroborating the registry in the same host of the present study, these authors showed a prevalence of 3.3% and an average intensity of 1.00. Similar results were obtained in the present study, The most recent record of parasitism was published by Cardoso et al. [11] in the coast of Pernambuco, northeast Brazil, in* Pseudupeneus maculatus* Bloch, 1793 with a prevalence of 8.3%, an average intensity of 1.1, and a variation of 1 or 2 parasites per fish. In the present survey, a lower prevalence of isopods was observed in the fish examined. The gill chamber was the preferred site of infestation for the parasite* R. signata*. The fish* L. jocu* is reported here as a new host for* R. signata*.

Specimens of* R. signata* had the same number of antenulla segments and antenna flagella as described by Brusca and Iverson [16] with 4 and 11 flagella segments respectively. In addition, this structure had the inverted “W” mark in the telson which is typical of this species. According to Pavanelli et al. [25] and Hermida et al. [24],* R. signata* is an isopod frequently found in fish in northeast Brazil. As other isopods,* R. signata *have a negative impact on fish's health. It induces slow growth and respiratory problems and cause injury to the host especially when large numbers of parasites are observed. In cases of severe parasitic infestation, opportunistic secondary fungal and bacterial infections may occur, which is most observed in confinement situations. However, because they are coastal fish, it was not possible to measure such disturbances, since parasite intensity and host immunity are also a determining factor for the occurrence of infections in aquatic populations.

*Cymothoa excisa* is a species of isopod that has low specificity, and in the world is already known occurring in several vertebrates, in fishes has been reported in 6 different families of fish including Lutjanidae [26, 27]. In Brazil the only record of this species of isopods in marine fish was made by Thatcher et al. [17] in* Micropogonias furnieri* (Sciaenidae) (Demarest, 1823). The occurrence of* C. excisa* in* L. analis* is in agreement with previous studies published by Weinstein [28] and Bunkley-Williams et al. [29]. These authors found the same parasite in the same species of fish and in the same infestation body site in hosts originated from the Caribbean Coast, Panama, and Bahia Portete, Colombia, respectively. This same isopoda had already been recorded in* Lutjanus*, in the Yucatan Peninsula, Mexico and Panama, where it was also recorded in three other species of Lutjanidae:* Lutjanus synagris*,* L. mahogoni* and* Ocyurus chrysurus* [19]. However, this parasite had not been found yet in* L. analis* in Brazil. Although they are protandritic animals, becoming functional males in one part of the cycle and later becoming functional females [19], according to Bonilla-Gómez et al. [26], isopods may be present as solitary parasites or parasites of both sexes may occur in a single host.

Allen [1] states that even* L. analis* and* L. jocu* which are both reef fish species, still have some differences regarding habitat:* L. analis* has preference for sandy bottoms with vegetation, estuaries, and regions near mangroves whereas* L. jocu* prefers coastal waters, particularly estuaries, and occasionally rivers.* C. excisa* was found only in* L. analis*, in this sense, Weinstein [28] explain that the habitat difference between fish species can contribute to the presence of isopoda.

The species* E. richardsoni* was also found only in male* L. jocu*. Barriga and Briones [30] reported the occurrence of isopods of the genus* Excorallana* in the east coast of Mexico; 7 species were identified:* E. acuticauda* Miers, 1881;* E. delaneyi* Stone and Heard, 1989;* E. oculata* Hansen, 1890;* E. subtilis* Hansen, 1890;* E. sexticornis* Richardson, 1901;* E. tricornis tricornis* Hansen, 1890; and* Excorallana* sp. Luque et al. (2013) reported* Excorallana* sp. on the body surface of* Ageneiosus inermis* Linnaeus, 1766, and* C. excisa* in the buccal cavity of* Micropogonias furnieri* Desmarest, 1823. To date, these are the only records of these two parasites of fish in Brazil. Koening [31] documented the occurrence of* Excorallana oculata *along the northeast coast of the country from the State Amapá to the State of Espírito Santo.

**Figure 6:**
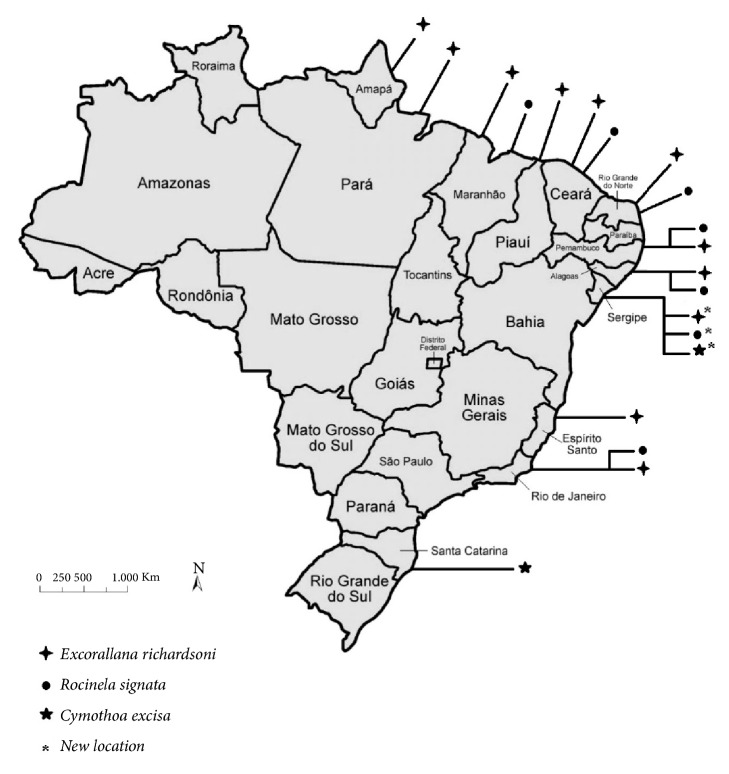
Map showing the distribution of the three species of isopods -* Rocinela siganta*,* Cymothoa excisa,* and* Excorallana richardsoni* in Brazil. The new location is highlighted. Source: Adapted and edited from Pinterest.

In Brazil,* E. richardsoni* is distributed in the States of Amapá, Pará, Maranhão, Ceará, Rio Grande do Norte, Pernambuco, Alagoas, Espírito Santo, and Rio de Janeiro. The first report of this isopod in the country was published by Koening and Coelho [32] in the State of Ceará. This parasite was found in samples collected during oceanographic expeditions. The author did not specify the host affected. The most recent record of this crustacean in Brazil was made by Silva and Souza-Filho [18] in the States of Amapá, Pará, Ceará, and Pernambuco. The parasite was found in samples from ships and expeditions along the north and northeast coast of Brazil between 1965 and 2000 (Figure 6).

In the present study, the morphological characteristics of* E. richardsoni* are in agreement with those presented by Silva and Souza-Filho [18]. Menzies and Kruczynski [15] describe that species of the genus* Excorallana* have bifid setae with mid-apical setae in the dorsal pleotelson region (Figure 4(d)). In our study, all the specimens analized presented the same setae bifid shape with a central midapical seta. However, one of the apical bristles was bifid (Figure 4(d), arrow). This characteristic is not mentioned in previous publications. This additional feature helps in the morphological description and taxonomic classification of these isopod species.

## 4. Conclusion

In conclusion, low levels of isopod infection were found in the fish species studied.* R. signata* presented the highest infection rates in* L. analis* and* L. jocu*. The presence of* E. richardsoni* and* R. signata *in the State of Sergipe, which lies on the northeast Brazil's Atlantic coast, may have occurred due to the migratory habit of the fish since this ectoparasites has been previously recorded in waters of the State Alagoas, northeastern region of the country, thus increasing its geographical distribution. Although the bifid end of the dorsal setae of the pleotelson is not a definitive morphological character used for the taxonomic identification of* E. richardsoni*, this particular feature was observed in all specimens examined in this study. The present survey presents the first report of three species of isopods in the state of Sergipe, the first record of* Cymothoa excisa* in* Lutjanus analis* in the country. We describe* Lutjanus jocu* as a novel host for* Rocinela signata* and* Excorallana richardsoni*.

## Figures and Tables

**Table 1:** Parasitological indices of Intensity, prevalence, and site of isopod infestation for *Lutjanus analis* and *Lutjanus jocu* in which: bc = buccal cavity; bf = branchial filaments; bs = body surface.

Host	Parasite	Prevalence(%)	Mean Intensity (parasites/fish)	Site of infestation
*Lutjanus analis*	*C. excisa*	1.44	2	bc
	*R. signata*	2.89	2	bf

*Lutjanus jocu*	*E. richarsoni*	21.05	8.25	bc, bf, bs
	*R. signata*	31.57	1.16	bf

## Data Availability

No data were used to support this study. The data of the present work comprise a part of the database of a doctoral research that is in progress, being not possible the release of this database of the research.
